# Supplementary material for: DIA-Based Quantitative Proteomics Reveals the Protein Regulatory Networks of Floral Thermogenesis in Nelumbo nucifera
Source: Int J Mol Sci. 2021 Jul 31;22(15):8251. doi: 10.3390/ijms22158251 (PMC8347412; doi:10.3390/ijms22158251)
Supplement: Supplementary file 1 [file ijms-22-08251-s001.zip › ijms-1311797-supplementary/Supplementary data/Supplementary figures.pdf]

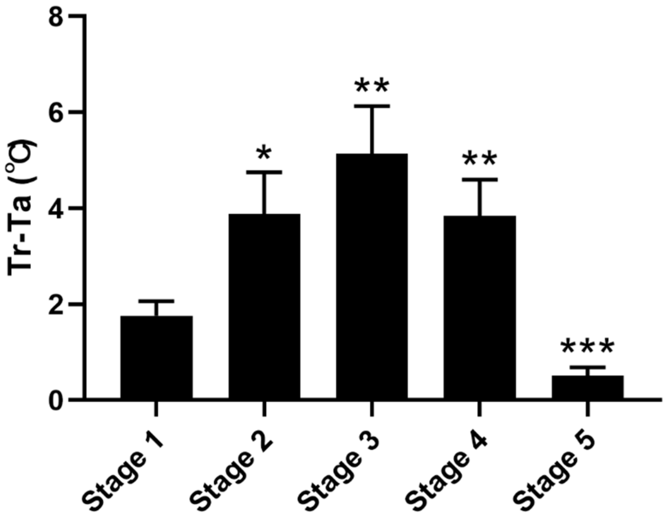

Figure S1. Thermogenesis pattern of *N. nucifera* receptacles at five developmental stages. The y-axis represents the difference between the receptacle temperature (Tr) and the ambient temperature (Ta). The error bars indicate the standard deviation of five replicates. Asterisks indicate a significant difference as determined by one-way ANOVA using stage 1 as a control (* $p < 0.05$; ** $p < 0.01$).

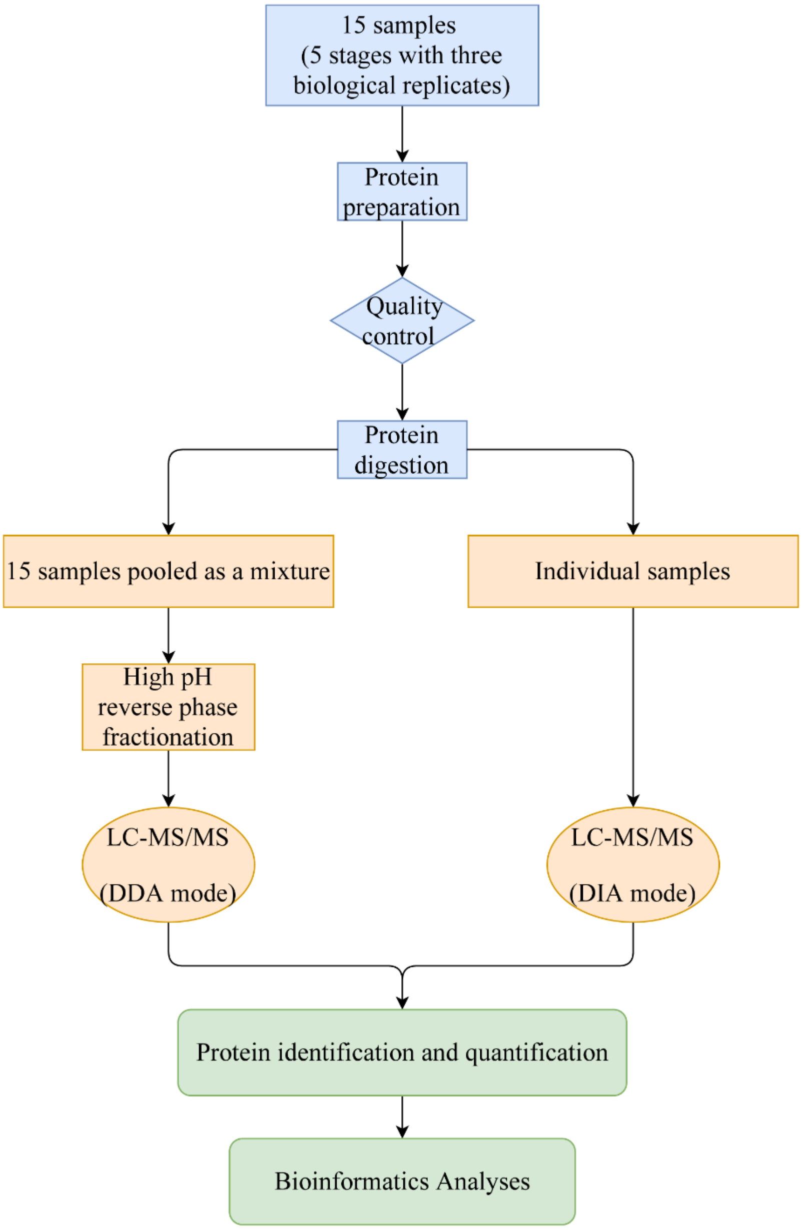

Figure S2. The experimental strategy of DIA-based mass spectrometry identification technology in the present study.

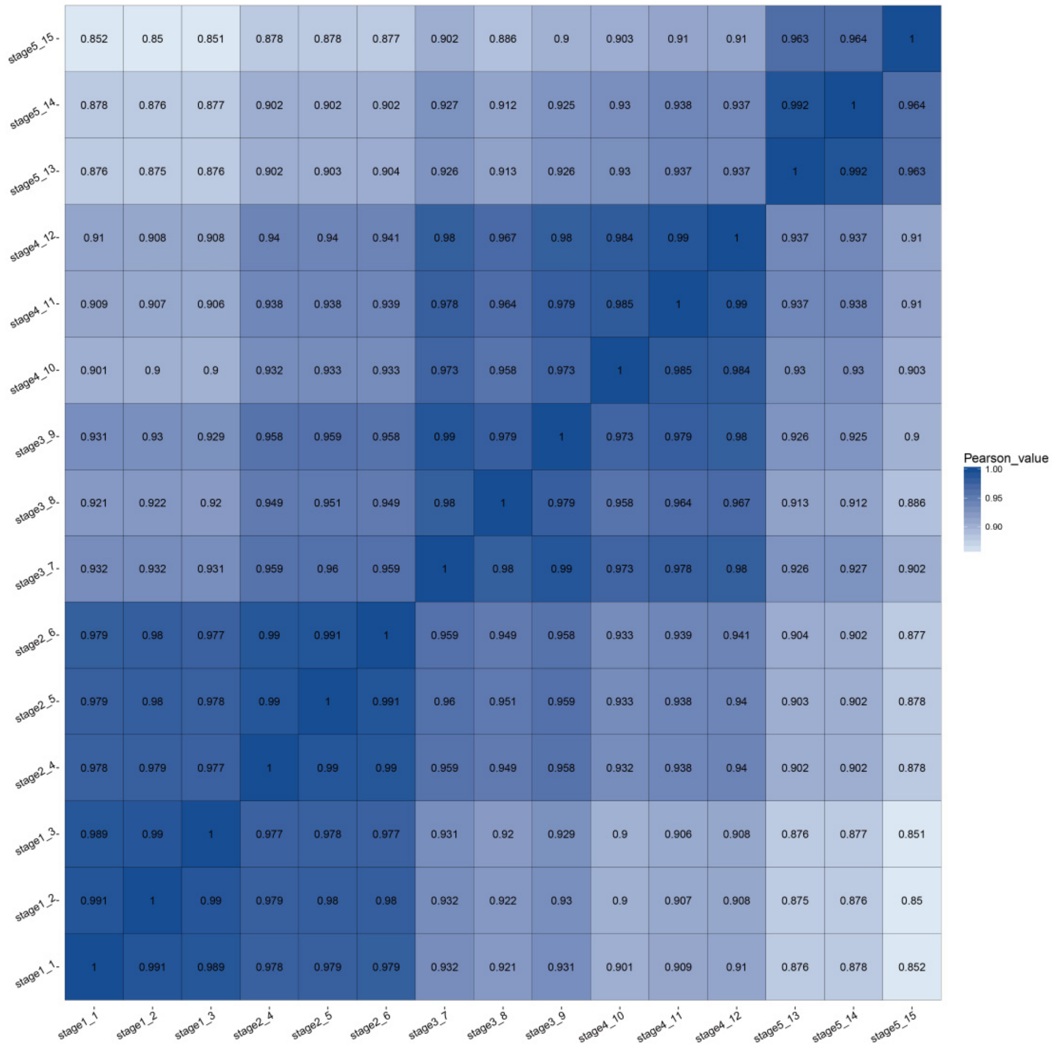

Figure S3. Heatmap of Pearson correlation between samples.

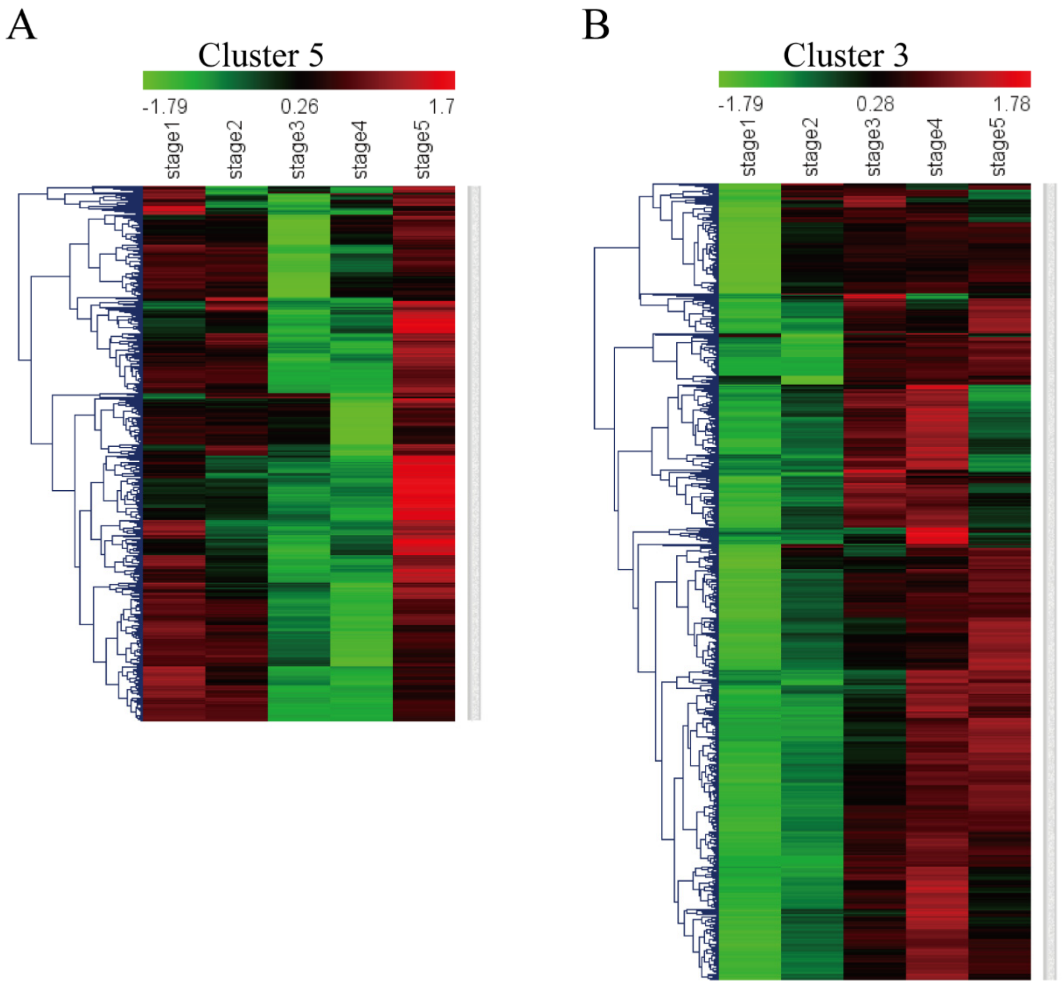

Figure S4. Heatmap of the abundance of proteins in cluster 5 (A) and cluster 3 (B).

A

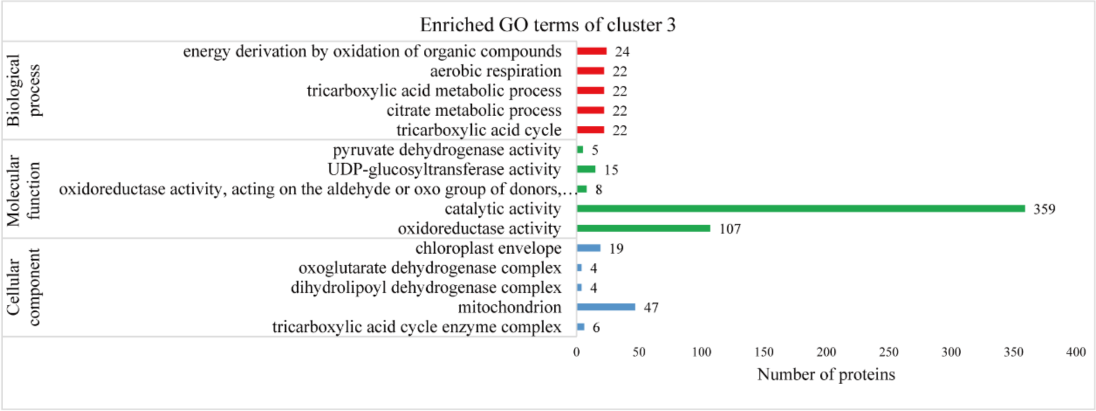

B

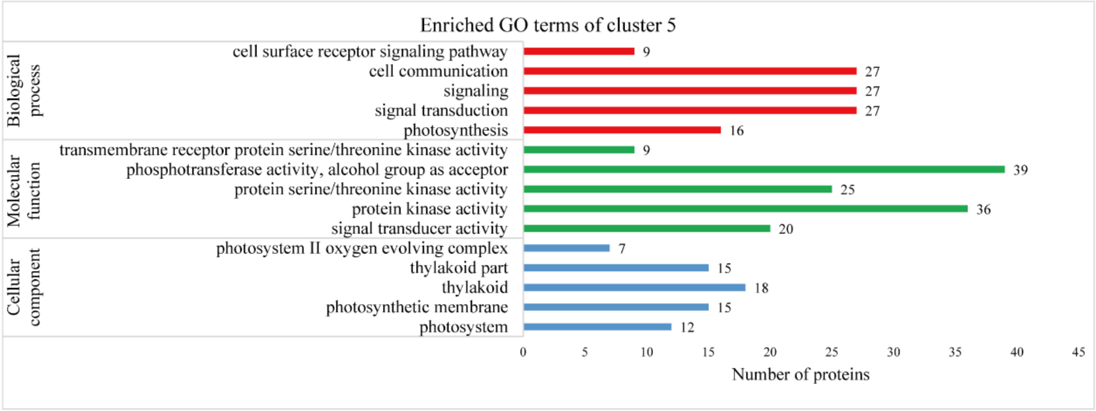

Figure S5. Top five enriched GO terms of cluster 3 (A) and cluster 5 (B). The abscissa represents the number of proteins in each GO term, and the ordinate represents the GO terms of three categories.

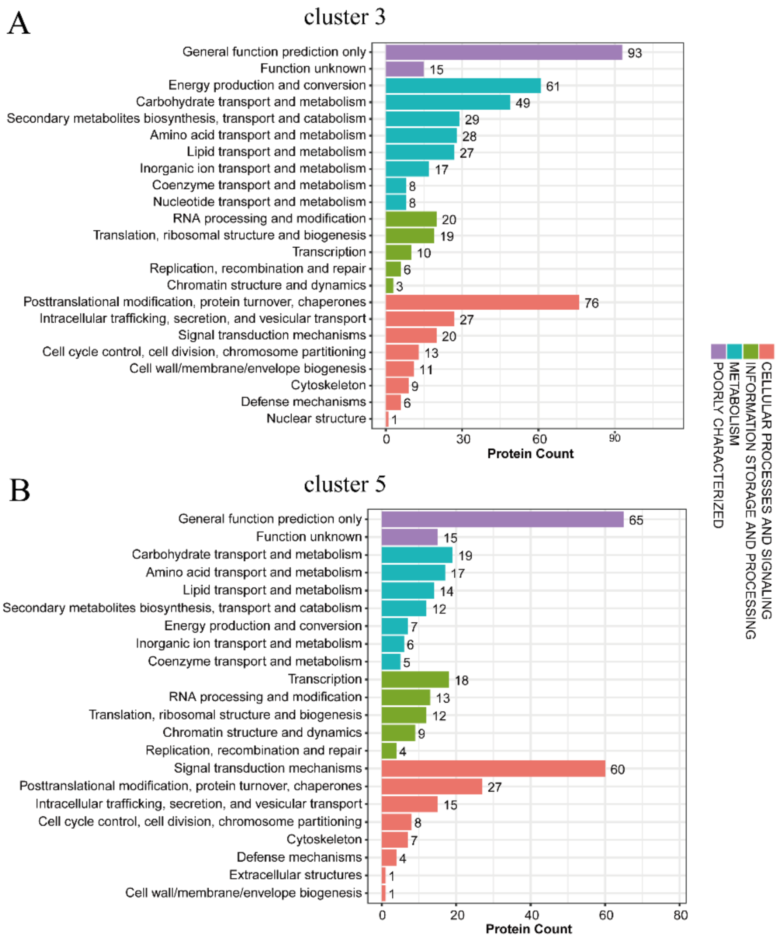

Figure S6. KOG annotation of the proteins in clusters 3 and 5.

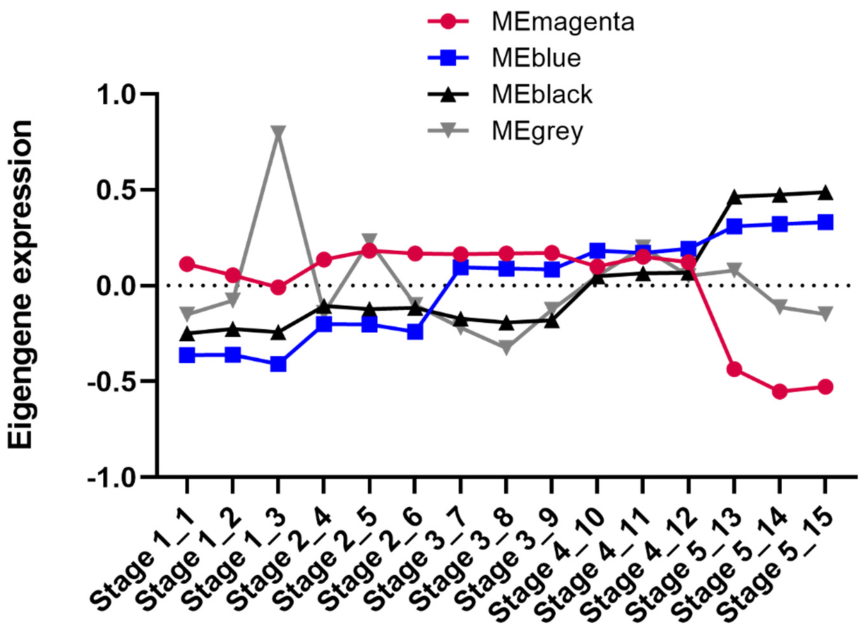

Figure S7. Temporal expression patterns of 'magenta', 'blue', 'black', and 'grey' modules.

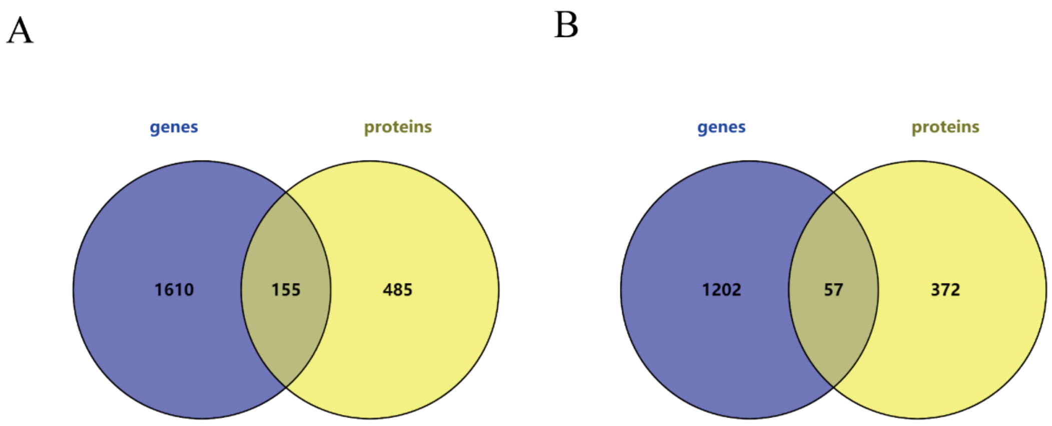

Figure S8. Omics correlation between transcriptome and proteome data. (A) DEGs/DAPs that were highly abundant during thermogenesis. (B) DEGs/DAPs that were low-abundant during thermogenesis.
